# Lipid Profiles and Obesity as Potential Risk Factors of Sudden Sensorineural Hearing Loss

**DOI:** 10.1371/journal.pone.0122496

**Published:** 2015-04-10

**Authors:** Joong Seob Lee, Dong Hyun Kim, Hyo Jeong Lee, Hyung Jong Kim, Ja Won Koo, Hyo Geun Choi, Bumjung Park, Sung Kwang Hong

**Affiliations:** 1 Department of Otorhinolaryngology-Head & Neck Surgery, Hallym University Sacred Heart Hospital, Anyang, Republic of Korea; 2 Department of Otorhinolaryngology-Head & Neck Surgery, Seoul National University Bundang Hospital, Seongnam, Republic of Korea; Nagoya University, JAPAN

## Abstract

**Objectives:**

The objective of our study was to establish whether increased lipid profiles and obesity affect the prevalence and prognosis of sudden sensorineural hearing loss (SSNHL).

**Methods:**

This was a case-controlled study with a longitudinal design. According to our criteria, 324 patients with SSNHL were included in this study. To manage potential covariates, 972 subjects with normal hearing from the Korean National Health and Nutrition Examination Survey were matched as control group according to their propensity scores. Age, level of total cholesterol (TC), high density lipoprotein cholesterol (HDL-C), low-density lipoprotein cholesterol (LDL-C), triglycerides (TG), and body mass index (BMI) were obtained from the clinical data. Multivariate logistic regression analysis was used to investigate the association between SSNHL and lipid profiles or obesity in the 1296 subjects. Multivariate Cox regression analysis was used to determine whether lipid profiles and obesity are prognostic factors in patients with SSNHL.

**Results:**

Mean body weight, BMI, TC, and TG were significantly higher in patients with SSNHL compared with control subjects (p<0.05). However, LDL-C values did not differ significantly between the two groups. Subjects with elevated TC and TG levels had a 2.20- (95% CI 1.50–3.24) and 1.50-fold (95% CI 1.08–2.08) increased odds, respectively, of SSNHL compared with subjects with normal TC and TG levels. Subjects with grade III BMI had a 1.59-fold (95% CI 1.17–2.16) increased odds of SSNHL. Multivariate Cox regression analyses revealed that BMI was an independent risk factor of treatment outcome, as patients with BMI ≥27.5 were less likely to achieve complete recovery than those with BMI <27.5 (p<0.05).

**Conclusions:**

The results of this study revealed that elevated TC and TG levels and increased BMI are significantly associated with the prevalence of SSNHL and its prognosis, indicating that vascular compromise may play an important role in the pathogenesis of SSNHL.

## Introduction

Sudden sensorineural hearing loss (SSNHL) is defined as hearing impairment of more than a 30 dB decrease, occurring over a 72 hour period, on three consecutive frequencies of pure tone audiometry [1]. Its incidence is estimated at 5–20/10,000 individuals per year [2,3].

Although various potential causes have been reported, including viral infections and immunologic diseases, [2,4–6] most cases of SSNHL are idiopathic. Recently, impairment of cochlear micro-vascular circulation has been proposed as the main etiology, given the hypothesis that the cochlea is supplied by a terminal artery without collateral circulation and is vulnerable to hypoxic damage [7]. Indeed, a few studies have suggested that microcirculation disturbance is clinically associated with the occurrence of SSNHL [8–12].

It is no wonder that vascular theory has been advocated as the main pathogenesis behind SSNHL because SSNHL, cardiovascular disease (CVD), and stroke are more likely to present in a similar fashion (ex: abruptly onset) in older age. However, other studies have reported that the vascular risk factors could not be responsible for auditory damage [6,13]. Thus, the association between SSNHL and vascular risk factors is still controversial, despite the biological plausibility.

Because no diagnostic modalities can efficiently evaluate cochlear micro-circulation in patients with SSNHL, it is difficult to fully investigate whether a change in plasma viscosity, such as that caused by hyperlipidemia, is associated with the occurrence and prognosis of SSNHL, unlike with CVD or stroke, where this association is easier to prove. Additionally, previous case-control studies about the risk factors of SSNHL have been criticized for selection bias because they were matched only for age and gender, neglecting other factors between the case subjects and matched controls [2,5,9]. Our main objective was to elucidate whether vascular risk factors such as lipid profiles may be associated with SSNHL and to investigate the role lipid profiles play in the prognosis of patients with SSNHL.

For this purpose, we conducted a large-scale matched case-control study using propensity scoring to reduce the selection bias and longitudinal study with a prospective component. Propensity score matching allows the distribution of the general baselines to be similar between case subjects and control subjects; thus, we expected that our study could demonstrate the role of lipid profiles as potential risk factor of SSNHL.

## Materials and Methods

Verbal informed consent protocol was used for the experimental group. The experimental subjects were provided with all pertinent information regarding purpose, procedure, potential benefits and alternatives to participation through a study information sheet, and then verbal agreement was obtained from those who wanted to participate in the study. We also recorded the verbal agreement in another study document. Our ethics committee (the Institutional Review Board of Hallym University Sacred Heart Hospital) approved this consent procedure because this was a large community-based study and some specific procedures or medications were not included for this study Although our study had a longitudinal prospective design, a chart review of the subjects who visited on a predetermined schedule after verbal agreement was also a major component of the study design

### Subjects and data source

This has two parts, a cross-sectional and a longitudinal design with a prospective component. 324 consecutive patients (162 males/162 females; mean age 49.64 years) diagnosed with unilateral SSNHL between January 2009 and February 2012 were enrolled as experimental group. Audiometric criterion of SSNHL was a rapid decrease in hearing of more than 30dB, affecting at least three consecutive frequencies. Pure tone threshold were obtained with pure tone at frequencies of 500, 1000, 2000, and 300 Hz. Exclusion criteria included 1) mixed hearing loss due to middle ear pathology, 2) prior history of sudden deafness or previous ear surgery, 3) presence of auditory nerve pathology such as vestibular schwannoma and 4) fluctuating hearing loss such as Meniere disease. All patients were hospitalized at the time of diagnosis of SSNHL. The serum concentrations of total cholesterol (TC), high density lipoprotein cholesterol (HDL-C), low-density lipoprotein cholesterol (LDL-C), and triglycerides (TG) were measured in all patients between 6 and 8 a.m. after an overnight fast. Body mass index (BMI) was calculated as weight in kilograms divided by height in meters squared (kg/m^2^).

Data for the control group were obtained from the Korean National Health and Nutrition Examination Survey (KNHANES) from 2009 to 2010. For each case, 3 subjects with normal hearing without middle ear pathologies (<25 dB) were matched as control subjects according to their propensity score to produce accurate results; thus, the test subjects and the control group were identical regarding all covariates such as gender, age, height and underlying diseases, except for lipid profiles (**Table 1**). Data of lipid profiles were obtained from fasting measurements of TC, HDL-C, and TG of KNHANES for control subjects. The LDL-C was calculated using the Friedewald calculation, which is valid when the TG level is ≤400 mg/dL. Therefore, our study only included patients with TG ≤400 mg/dL. BMI was calculated in the same manner. The control group included 972 individuals (452 males/520 females; mean age 48.78 years) (**Table 1**).

**Table 1:** Baseline characteristics of the test subjects and the control group after propensity score matching.

	SSNHL (n = 324)	Control (n = 972)
Age, years (s.d.)	49.64±16.52	48.78±14.70
Female, n (%)	162 (50)	520 (53.5)
Height, cm (s.d.)	162.3±9.52	162.4±8.99
Hypertension, n (%)	71 (21.9)	175 (18)
Diabetes, n (%)	41 (12.6)	86 (8.9)
Heart disease, n (%)	6 (1.9)	17 (1.8)
Stroke, n (%)	8 (2.5)	13 (1.3)

Values are expressed as means (s.d.)

The test subjects and the control group were not statistically different regarding all covariates such as gender, age, height and underlying diseases.

For longitudinal analysis, all patients with SSNHL (n = 324) were hospitalized and administered 60 mg of oral prednisolone for 7 days. After discharge, patients underwent follow-up audiometry in addition to a clinical examination at predetermined intervals (2 weeks, 1, 2, 4, and 6 months, then yearly). Subjects were censored on the day of complete recovery (CR, final hearing level ≤25 dB) according to Siegel’s criteria, [14] or at the final follow-up. Exclusion criteria included 1) presence of ischemic heart disease or renal failure, and 2) prior history of stroke. Patients who declined to participate, were lost to follow-up or showed fluctuating hearing loss were also excluded during follow up days. Thus, 295 of 324 patients with SSNHL were included in this longitudinal analysis of the prognostic value of lipid parameters in SSNHL.

### Outcome measures

The optimal ranges of lipid parameters were defined as TC ≤200 mg/dL, LDL-C ≤130 mg/dL, TG ≤150 g/dL, and HDL-C ≥60 mg/dL, according to guidelines of the American Association of Clinical Endocrinologists (AACE) [15]. BMI levels were divided into three groups according to the recommendations of the World Health Organization [16]: 1) less than 23.0 kg/m^2^, 2) 23.0–27.5 kg/m^2^, and 3) 27.5 kg/m^2^ or higher. Patients with SSNHL were divided into CR or non-CR groups according to their final audiogram.

### Statistical analysis

Based on the calculated propensity score using logistic regression according to their covariates including gender, age, height and underlying disease, each patient with SSNHL was matched with three individuals with normal hearing from the KNHANES according to an optimal algorithm (case-control matching with the same propensity score) using Excel. Subsequently, all subjects were subcategorized into groups according to age, levels of TC, LDL-C, HDL-C, TG, and BMI. Multivariate logistic regression analysis was used to investigate the association between SSNHL and lipid profiles. Odd ratios (ORs) are provided with 95% confidence intervals (CI).

Multivariate Cox regression analysis was used to determine the significance of lipid profiles in the prognosis of SSNHL. The follow-up period was defined as the length of time from the onset day of SSNHL to CR according to Siegel’s criteria and censoring due to another cause. All statistical analyses were two-tailed and a *p* value of less than 0.05 was considered statistically significant. The statistical calculations were performed using the SPSS software program, version 18.0 for Windows (SPSS Inc., Chicago, IL, USA).

## Results

### Demographic findings

Overall, 324 patients with SSNHL and 972 with normal hearing from the KNHANES data were included in our study. Table 1 lists the demographic characteristics of the subjects. The underlying covariates after propensity score matching were similar between the two groups except for lipid parameters. Mean body weight and BMI were calculated at 63.8±11.55 kg and 23.91±3.29, respectively, for patients with SSNHL, and 61.7±11.14 kg and 23.30±3.21 for control subjects. Mean body weight and BMI were significantly higher for patients with SSNHL compared with control subjects (*p*<0.05). TC and TG values were calculated at 192.82±37.9 mg/dl and 122.78±77.21 mg/dl, respectively, for patients with SSNHL, and 183.46±34.89 mg/dl and 109.63±65.32 mg/dl for control subjects. TC and TG levels were also significantly higher in patients with SSNHL compared with control subjects (*p*<0.05). LDL-C values did not differ significantly between the two groups, but HDL-C values were significantly higher in patients with SSNHL compared with the control subjects (**Table 2**).

**Table 2:** Serum lipid profiles and obesity in SSNHL patients and matched controls.

	SSNHL (n = 324)	Control (n = 972)	P
Body weight (Kg)	63.8± 11.55	61.7 ± 11.14	0.005
BMI (kg/m^2^)	23.91 ± 3.29	23.30 ± 3.21	0.004
TC (mg/dl)	192.82 ± 37.9	183.46 ± 34.89	< 0.001
HDL (mg/dl)	57.62 ±15.3	54.34 ±13.05	<0.001
LDL (mg/dl)	110.65 ±35.61	107.21±31.25	0.122
Triglyceride (mg/dl)	122.78±77.21	109.63 ±65.32	0.003

BMI, Body mass index; TC, total cholesterol; HDL, high density lipoprotein; LDL, low-density lipoprotein; TG, triglycerides Values are expressed as means (s.d.).

### Association between SSNHL prevalence and lipid profiles

The association between abnormal vascular risk factors and the prevalence of SSNHL was examined in 1296 subjects using multivariate analysis. Subjects with abnormal TC and TG levels were 2.20- (95% CI 1.50–3.24) and 1.50-fold (95% CI 1.08–2.08), respectively, odds more likely to have SSNHL than those with normal TC and TG levels. Regarding BMI, subjects with a grade III BMI had a 1.59-fold (95% CI 1.17–2.16) increased odds of SSNHL compared with those with a grade I or II BMI (**Table 3**).

**Table 3:** Analysis of lipid profiles as risk factors for sudden sensorineural hearing loss by multivariable logistic regression.

		Multivariate analysis ORs (95%CI)	P
**Age (years)**	<21	1	
	21–40	0.462 (0.243–0.879)	0.019
	41–60	0.412 (0.219–0.776)	0.006
	61–80	0.457 (0.235–0.887)	0.021
	≥81	2.498 (0.609–10.242)	0.203
**TC (mg/dL)**	Normal (≤200)	1	
	Abnormal (>200)	2.202 (1.497–3.240)	<0.001
**HDL (mg/dL)**	Normal (≥60)	1	
	Abnormal (<60)	0.762 (0.566–1.025)	0.073
**TG (mg/dL)**	Normal (≤150)	1	
	Abnormal (>150)	1.496 (1.076–2.080)	0.017
**LDL (mg/dL)**	Normal (≤130)	1	
	Abnormal (>130)	0.811 (0.534–1.232)	0.326
**BMI**	Grade I (<23.5)	1	
	Grade II (<27.5)	1.199 (0.855–1.682)	0.293
	Grade III (≥27.5)	1.587 (1.167–2.159)	0.003

TC, total cholesterol; HDL, high density lipoprotein; TG, triglycerides; LDL low-density lipoprotein; BMI, Body mass index; ORs: Odds ratios.

### Lipid parameters as treatment-outcome factors of SSNHL

In total, 295 patients with SSNHL were included in this study to assess the prognostic value of lipid profiles in SSNHL. The mean follow-up period was 11±19.72 weeks (1–200 weeks). The mean±2 standard deviations of the initial average hearing level of the involved ear was 69.98±24.11 dB (range 18–120 dB) and the last average hearing level was 42.72±31 dB (range 0–120 dB). CR was achieved in 107 patients (36.3%, 107/295) during 3245.2 weeks of patient follow-up.

Multivariate analysis revealed that higher BMI and increased age were both associated with increased risk of CR failure (**Table 4**). In particular, it revealed a dose–response relationship between age and CR failure rate. CR failure rate in patients aged 41–60, and 61 and older, were 3.34 (95% CI: 1.12–10) and 10.98 (95% CI: 3.11–38.74) times higher, respectively, than in patients aged 40 and younger. The CR failure rate in patients with BMI ≥27.5 was 1.75 times higher (95% CI: 1.00–3.076) than in those with BMI <27.5.

**Table 4:** Multivariable logistic regression analysis for outcome (treatment failure) among patients with sudden sensorineural hearing loss.

		Multivariate analysis ORs (95%CI)	P
**Age (years)**	<21	1	
	21–40	1.937 (0.644–5.823)	0.239
	41–60	3.340 (1.116–9.997)	0.031
	≥61	10.976 (3.110–38.79)	<0.001
**TC (mg/dL)**	Normal (≤200)	1	
	Abnormal (>200)	1.198 (0.592–2.424)	0.615
**HDL (mg/dL)**	Normal (≥60)	1	
	Abnormal (<60)	0.946 (0.519–1.723)	0.856
**TG (mg/dL)**	Normal (≤150)	1	
	Abnormal (>150)	0.769 (0.431–1.372)	0.374
**LDL (mg/dL)**	Normal (≤130)	1	
	Abnormal (>130)	0.589 (0.276–1.258)	0.172
**BMI**	Grade I-II (<27.5)	1	
	Grade III (≥27.5)	1.754 (1.000–3.076)	< 0.050

TC, total cholesterol; HDL, high density lipoprotein; TG, triglycerides; LDL low-density lipoprotein; BMI, Body mass index; ORs: Odds ratios.

**Fig 1:**
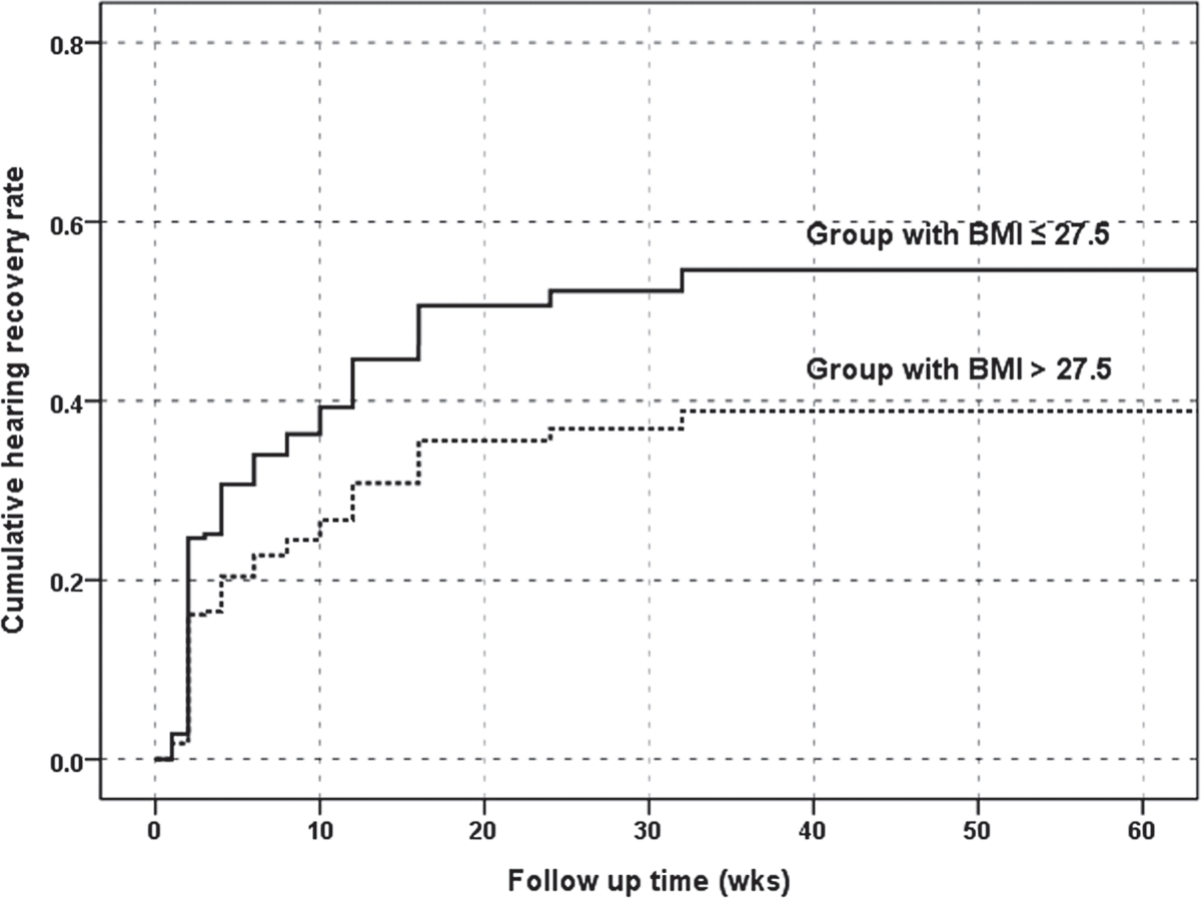
Cumulative complete hearing recovery rate according to body mass index (BMI). Cumulative complete recovery rate between subjects with body mass index (BMI) equal to or greater than grade III, and those with BMI less than grade II. The hazard ratio of poor response with patients with BMI equal or higher than grade III was 1.606 (95% confidence interval: 1.03–2.50).

Multivariate Cox regression analysis revealed that high BMI was an independent risk factor of treatment outcome, indicating that patients with BMI ≥27.5 (grade III) were less likely to achieve CR than were those with BMI <27.5 (*p*<0.05) (**Fig 1**).

## Discussion

The results of our study revealed that cardiovascular risk profiles such as elevated TC and TG levels and increased BMI are significantly associated with the prevalence of SSNHL and its prognosis. Because the cochlea is very vulnerable to ischemic injury and lacks collateral circulation besides the cochlear artery (supplied by the labyrinthine artery), blood viscosity elevations due to hyperlipidemia can disturb the cochlear microcirculation. Thus, blood lipid level has been suggested as a main risk factor of SSNHL as well as CVD [2,6,8]. However, many studies of the association between SSNHL and lipid profiles are inconclusive, with often contradictory results [8,12,17,18]. Furthermore, some studies matched a flawed control group to justify their results without considering the full range of covariates between case subjects and matched controls [3,6,9,11,17].

Propensity score matching is being increasingly used to reduce the effects of outcome-selection bias in the estimation of causal risk factor effects using observational data [19]. Many previous studies have demonstrated that matching on the propensity score could eliminate a baseline difference between case subjects and controls [20,21]. The major strength of our study is that a large number of subjects from a national database were matched with control subjects using propensity scores, which allowed adjustments for different baseline characteristics including age, gender, height, and underlying disease between case subjects and matched control subjects (**Table 1**). We believe that this case-control matching facilitated accurate estimation of the impact of the lipid profile in SSNHL, so our study has the distinct characteristic of providing high-quality evidence.

We found that values for TC, TG, and BMI tended to be higher among patients with SSNHL than among healthy subjects. TC was calculated as a summation of HDL-C, LDL-C, and TG, and the results indicate that SSNHL is critically associated with a high TG level. Patients with a high TG level (>150 mg/dl) had a 1.50-fold increased odds of SSNHL, with statistical significance (**Table 3**).

Previous studies have reported that classic risk factors of cardiovascular disease such as high TC and LDL-C levels are significantly associated with SSNHL [5,9,22]. They have speculated that a high LDL-C level could lead to endothelial damage in peripheral arteries, thereby leading to SSNHL due to disturbance of circulation in the cochlear end artery. In contrast, our results revealed that TG is an independent risk factor for SSNHL, while the LDL-C level was not associated with SSNHL.

TG level is not generally considered a major risk factor for cardiovascular disease despite abundant clinical evidence to the contrary. Furthermore, AACE Guidelines do not refer to a definite benefit of lowering TG levels to prevent CVD [15]. However, considering that isolated TG enrichment sufficiently induces alteration of cholesterol delivery and removal mechanisms, abnormalities in which may contribute to damaged endothelial function, [23] TG enrichment could also lead to disturbances in cochlear microcirculation.

Interestingly, obesity was also significantly associated with SSNHL. This is, to our knowledge, the first report to study the association between BMI and SSNHL. Patients with BMI ≥27.50 (grade III according to the index for the Asian population) have a 1.59-fold increased odds for prevalence of SSNHL (95% CI: 1.17–2.16). A previous study reported that BMI is directly associated with TG, but found no association between BMI and LDL [24]. Our results revealed that abnormal TG enrichment and a high BMI were associated with SSNHL, which corresponds well with those of a previous study [24]. However, considering that BMI and LDL are well-established risk factors for CAD, [15] and assuming that our vascular theory is correct, the lack of association between the LDL level and SSNHL remains questionable and should be investigated in further studies.

We investigated the influence of lipid profiles on the prognosis of SSNHL using longitudinal analysis. During 3245.2 weeks of patient follow-up, 107 patients (36%) achieved CR on pure tone audiometry according to Sigel’s criteria [14]. We found that BMI and age were associated with the prognosis of SSNHL on multivariate analysis like as CVD. Patients with BMI ≥27.5 (grade III) had a 1.75-fold increased risk (95% CI: 1.00–3.076) of failure to reach CR compared to those with BMI <27.5. ORs of failure in CR in patients aged 41–60, and 61 and older, were 3.34 (95% CI: 1.12–10) and 10.98 (95% CI: 3.11–38.74), respectively, so individuals aged >41 had a poorer response than younger individuals (**Table 4**). However, other lipid parameters and presence of DM or HTN did not significantly affect SSNHL prognosis (data not shown).

Multivariate Cox regression analysis revealed that BMI was an independent risk factor of treatment outcome during follow up, indicating that patients with BMI ≥27.5 were less likely to achieve CR after SSNHL compared with those with BMI <27.5 which is a novel finding (*p*<0.05) (**Fig 1**).

In conclusion, our results indirectly support the vascular theory of SSNHL development. We demonstrated that TG, TC, and BMI are associated with the prevalence of SSNHL and its prognosis. We believe that propensity score matching in our large-scale study allows a high degree of confidence. Nevertheless, our results should be substantiated with further evidence-based research for the following reasons. First, a cross-sectional model is unsuitable for estimating the predictive value of lipid parameters in SSNHL. Second, adjustment for important unbalanced factors that could confound the association, such as smoking, lipid lowering drug use, aspirin use, and NSAID use was not considered owing to the difficulty of data collection. However, considering that BMI was an independent prognostic factor in our longitudinal study, it is quite apparent that lipid profiles may be associated with the incidence and prognosis of SSNHL.
